# Effect of Ferulic Acid Loaded in Nanoparticle on Tissue Transglutaminase Expression Levels in Human Glioblastoma Cell Line

**DOI:** 10.3390/ijms25158397

**Published:** 2024-08-01

**Authors:** Paola Dell’Albani, Claudia Carbone, Giovanni Sposito, Michela Spatuzza, Maria Assunta Chiacchio, Rosaria Grasso, Laura Legnani, Debora Santonocito, Carmelo Puglia, Rosalba Parenti, Giovanni Puglisi, Agatina Campisi

**Affiliations:** 1 Institute for Biomedical Research and Innovation, CNR, Via P. Gaifami, 18, 95126 Catania, Italy; paola.dellalbani@cnr.it (P.D.); michela.spatuzza@cnr.it (M.S.); 2 Department of Drug Sciences and Health, University of Catania, 95125 Catania, Italy; ccarbone@unict.it (C.C.); giovanni.sposito@unict.it (G.S.); ma.chiacchio@unict.it (M.A.C.); debora.santonocito@unict.it (D.S.); capuglia@unict.it (C.P.); puglisig@unict.it (G.P.); 3 NANOMED, Research Center on Nanomedicine and Pharmaceutical Nanotechnology, University of Catania, 95125 Catania, Italy; 4 CERNUT, Research Centre for Nutraceuticals and Health Products, University of Catania, 95125 Catania, Italy; 5 Oasi Institute for Research on Mental Retardation and Brain Aging (IRCCS), 94018 Troina, Italy; 6 Department of Physics and Astronomy “Ettore Majorana”, University of Catania, 95123 Catania, Italy; rosaria.grasso@ct.infn.it; 7 Department of Biotechnology and Biosciences, University of Milan-Bicocca, 20126 Milan, Italy; laura.legnani@unimib.it; 8 Department of Biomedical and Biotechnological Sciences, Section of Physiology, University of Catania, 95123 Catania, Italy; parenti@unict.it

**Keywords:** tissue transglutaminase (TG2), ferulic acid, glioblastoma, human glioma cell line, nanostructured lipid carrier (NLC), cell cycle progression

## Abstract

Glioblastoma (GBM) is one of the most aggressive cancers, characterized by a decrease in antioxidant levels. Evidence has demonstrated that ferulic acid (FA), a natural antioxidant particularly abundant in vegetables and fruits, could be a promising candidate for GBM treatment. Since FA shows a high instability that compromises its therapeutic application, it has been encapsulated into Nanostructured Lipid Carriers (NLCs) to improve its bioavailability in the brain. It has been demonstrated that tissue transglutaminase (TG2) is a multi-functional protein implicated in many physiological and pathological processes, including cancer. TG2 is also involved in GBM correlated with metastasis formation and drug resistance. Therefore, the evaluation of TG2 expression levels and its cellular localization are important to assess the anti-cancer effect of FA against GBM cancer. Our results have demonstrated that treatment with free FA and FA-NLCs in the U87-MG cancer cell line differently modified TG2 localization and expression levels. In the cells treated with free FA, TG2 appeared expressed both in the cytosol and in the nucleus, while the treatment with FA-NLCs showed that the protein is exclusively localized in the cytosol, exerting its pro-apoptotic effect. Therefore, our data suggest that FA loaded in NLCs could represent a promising natural agent for supplementing the current anti-cancer drugs used for the treatment of GBM.

## 1. Introduction

Glioblastoma (GBM) is the most aggressive and common form of adult primary brain cancer [1,2,3], characterized by resistance to chemotherapy and radiation, decreased antioxidant levels, and exhibiting a high propensity for recurrence [4]. Evidence has demonstrated that tissue transglutaminase (TG2) plays a key role in GBM cell biology [5], and it is correlated with metastasis formation and the overall poor survival of patients. TG2 is a multi-functional calcium-dependent protein that catalyzes cross-linking reactions and can bind and hydrolyze guanosine-5′-triphosphate (GTP) [6]. TG2 can also function as a G protein, a kinase [7,8], and a protein disulfide isomerase [9]. It is present in the extracellular matrix, plasma membrane, cytosol, mitochondria, and nucleus [10]. TG2, depending on cell localization, calcium (Ca^2+^) concentration, and GTP levels, plays different roles [11]. When Ca^2+^ levels are low and GTP levels are high, TG2 exhibits a “closed” conformation and is predominantly localized in the nucleus, playing a role in controlling cell proliferation, regulating gene expression, and promoting cell survival [12]. In contrast, when Ca^2+^ levels are high and those of GTP are low, TG2 shows an “open” conformation and is prevalently localized in the cytosol, activating the apoptotic pathway and inducing cell death [13]. In recent years, researchers’ attention has been focused on natural compounds as potential therapeutic agents for GBM [14,15,16,17,18,19].

**Figure 1:**
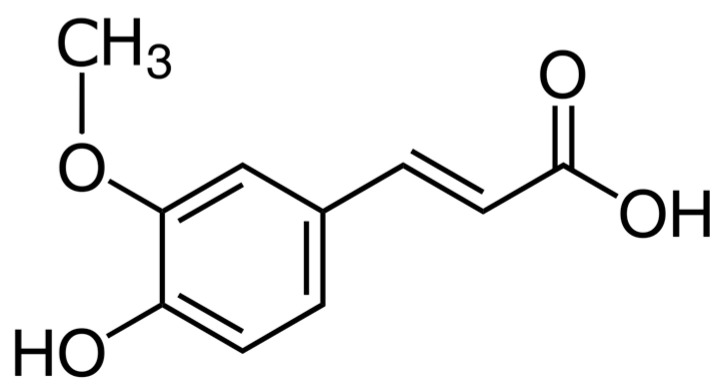
Chemical structure of ferulic acid.

In our previous study, we analyzed, on a human glioblastoma cell line, the U87-MG, the effect of the Ferulic Acid (FA) from *Ferula foetida* L. [20], a natural antioxidant particularly abundant in vegetables and fruits, known to exert anti-cancer effects [21] (Figure 1).

We demonstrated that FA was able to induce a synergic pro-apoptotic effect on U87-MG, which was activated by its loading on Nanostructured Lipid Carriers (NLCs) [22,23,24], showing the important role of drug encapsulation. Notwithstanding this interesting property, the potential therapeutic property of FA is strongly compromised due to its low water solubility, inability to cross lipophilic barriers, and extensive first-pass metabolism [25]. Therefore, we used NLCs that represent an innovative strategy to overcome the limits of FA in order to allow for the potential application in the therapeutic field [26]. In particular, NLCs have small-sized particles and are highly homogeneous and stable, showing good systemic tolerability, high efficiency of drug encapsulation, and controlling release properties without any cytotoxic effect [27].

Herein, we assessed the effect of free FA, FA-loaded NLCs (FA-NLC), and blank NLCs on TG2 localization and expression levels in a U87-MG glioma cell line. Furthermore, treated U87-MG were analyzed to detect the TG2 kinase activity, which plays a key role in regulating the cell cycle progression, and un-phosphorylated/phosphorylated retinoblastoma (Rb) proteins. Moreover, p53 and cyclin D_1_ expression levels, proteins involved in cell cycle progression, were also analyzed. In addition, to verify if the treatment with free FA, FA-NLCs, and blank NLCs was able to activate the pro-apoptotic activity of TG2, PARP-1 and caspase-3 cleavage was evaluated.

## 2. Results

### 2.1. Physico-Chemical, Technological, and Morphological Characterization of Blank NLCs and FA-NLCs

**Figure 2:**
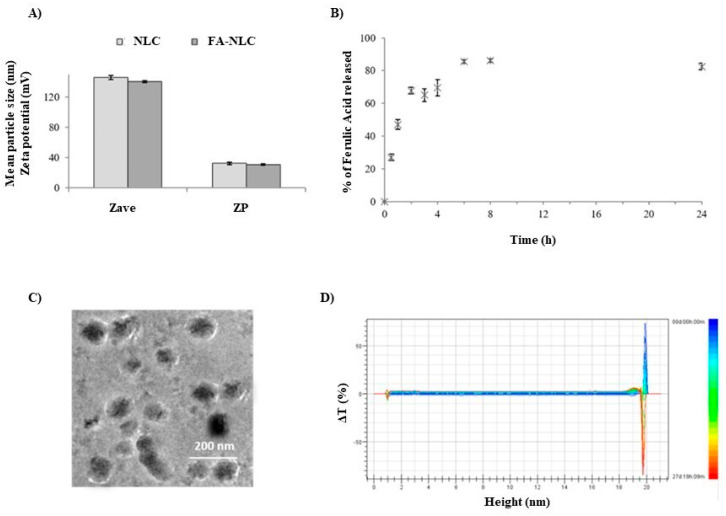
Physical–chemical characterization of FA-NLCs. (**A**) Mean particle size (Z-Ave) and Zeta potential (ZP) compared to blank NLCs. (**B**) Percentage of FA released after 24 h. (**C**) Cryo-TEM image. (**D**) Delta transmission profile obtained through Turbiscan for sample stored at 25 °C for 27 days.

Figure 2 reports the technological characterization of blank NLCs and FA-NLCs. Both nanoformulations presented a small and homogeneous particle size lower than 150 nm (Figure 2A). This observation is supported by Cryo-TEM analysis showing a positive zeta potential (≈+30 mV), which predicts good long-term stability of the nanoformulations (Figure 2C). This behavior, as confirmed by Turbiscan Stability studies, was performed at 25 °C for 27 days. The linearity of the transmission profiles (ΔT) clearly demonstrates physical stability during storage, without the occurrence of particle aggregation or migration at the top or at the bottom of the cuvette (Figure 2D). Furthermore, NLCs were able to bind FA and to release it in a modulated way for 24 h (Figure 2B). In particular, in our previous studies we found that FA is released immediately within 2 h and is subsequently released slowly (over 80%), when it is encapsulated in the lipid matrix [26]. In addition, we demonstrated through CLSM analysis in U87-MG cultures that the coating layer of the cationic lipid of NCLs was able to improve cellular uptake [27].

### 2.2. Effects of Blank NLCs, Free FA, and FA-NLCs on the Percentage of Cellular Viability

**Figure 3:**
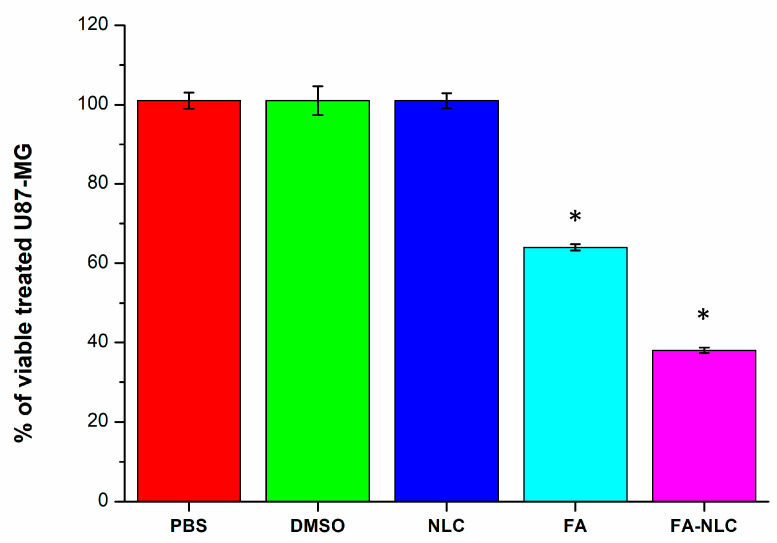
Percentage of cellular viability in U87-MG cell line cultures exposed for 24 h to 0.01% PBS, 0.01% DMSO, blank NLCs, 36 µM free FA, and 36 µM FA-NLCs. Results represent the mean ± SD of n = 4 separated experiments performed in triplicate. Statistical analysis is evaluated through one-way ANOVA test. * *p* < 0.01 significant differences vs. PBS and DMSO.

The effect of blank NLCs, 36 µM free FA, and 36 µM FA-NLCs on the percentage of the cellular viability in U87-MG cell line cultures after 24 h treatment was evaluated (Figure 3). The experiments were performed to have a final concentration of 36 µM of the percentage of FA and FA-NLCs, as reported in previous studies. In addition, we found that the optimal exposure time of U87-MG cell cultures to blank NLCs, FA, and FA-NLCs was 24 h [22,28]. Data were expressed as the percentage of cellular viability, and the significance was evaluated in comparison with PBS and DMSO-treated cells, used as controls. No significant differences between PBS and DMSO-treated U87-MG cells were found; therefore, both groups were used as control. The treatment with blank NLCs did not modify the percentage of cellular viability when compared with the controls, while the exposure of the cells to free FA induced a significant decrease in the percentage of cell viability when compared with the control. Furthermore, when the cells were treated with FA-NLCs, a strong reduction (~60%) in the percentage of cellular viability was found.

### 2.3. Effects of Blank-NLCs, Free FA, or FA-NLCs on TG2 Localization and Expression Levels

**Figure 4:**
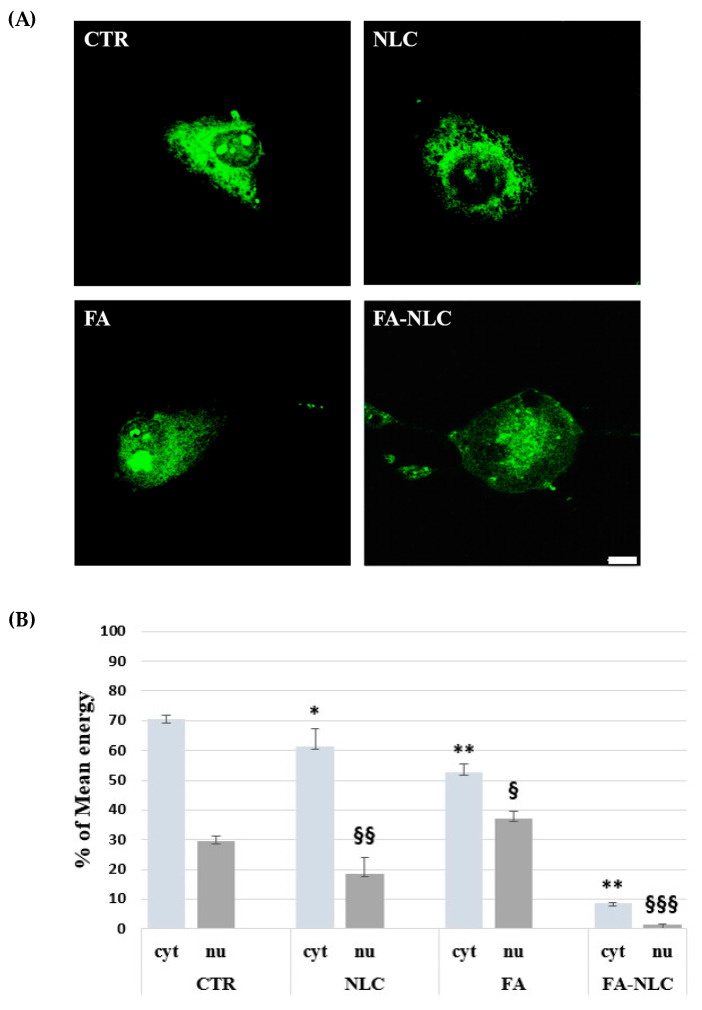
(**A**) The panel shows CLSM images of U87-MG stained with TG2 antibody in control condition (CTR) and after 24 h treatment with blank NLCs (NLC), 36 µM free FA (FA), and 36 µM FA-NLCs (FA-NLC). Cells were incubated with mouse monoclonal antibody against TG2 and then with FITC-conjugated polyclonal antibody. Scale bar = 10 µm. (**B**) Histogram represents the CLSM mean energy of cytoplasmatic (cyt) and nuclear (nu) positivity for TG2 in the different experimental conditions. Data are expressed as the mean energy ± S.D. of the values obtained from 10 cells of the values of n = 3 independent experiments performed in triplicate. Statistic was performed through two-way ANOVA. * *p* = 0.017 CTR (cyt) vs. NLC (cyt); ** *p* < 0.001 CTR (cyt) vs. FA (cyt) and FA-NLC (cyt); § *p* = 0.04 CTR (nu) vs. FA (nu); §§ *p* = 0.003 CTR (nu) vs. NLC (nu); §§§ *p* < 0.0001 CTR (nu) vs. FA-NLC (nu).

To assess the intracellular localization and the expression levels of TG2 in U87-MG treated with blank NLCs, 36 µM free FA, and 36 µM FA-NLCs for 24 h, CLSM acquisition and Western blotting analysis were used. For each CLSM imaging acquisition of immunofluorescence single-channel for TG2 intracellular localization on a single cell was performed. CLSM acquisition showed no significant differences in cell morphology in the control (Figure 4A). TG2 appeared prevalently localized at high levels in the cytosol, in the nucleus, and in the nucleoli. The treatment of the U87-MG cell cultures with blank NLCs induced a significant reduction in TG2 levels when compared with the control. The protein appeared expressed at low levels both in the cytosol and in the nuclear compartment. In addition, cells showed a morphology similar to that one observed in the control (Figure 4A). When the cells were exposed to free FA, TG2 appeared at lower levels and in a “diffused manner” in the cytosol, in the nucleus, and nucleoli when compared with the control. In addition, the cells appeared to be suffering and showed an amoeboid aspect (Figure 4A). The treatment with FA-NLCs dramatically affected the positivity of the cells for TG2, which showed a strong stain in the cytoplasm. In addition, the protein was absent in the nucleus and in the nucleoli (Figure 4A). Furthermore, CLSM mean energy of cytoplasmatic (cyt) and nuclear (nu) positivity for TG2 in the different experimental conditions was performed through ImageJ Software (version 2.0.0-rc-69, 1.52p, free software downloaded using the Image Calculator function) (Figure 4B). 

**Figure 5:**
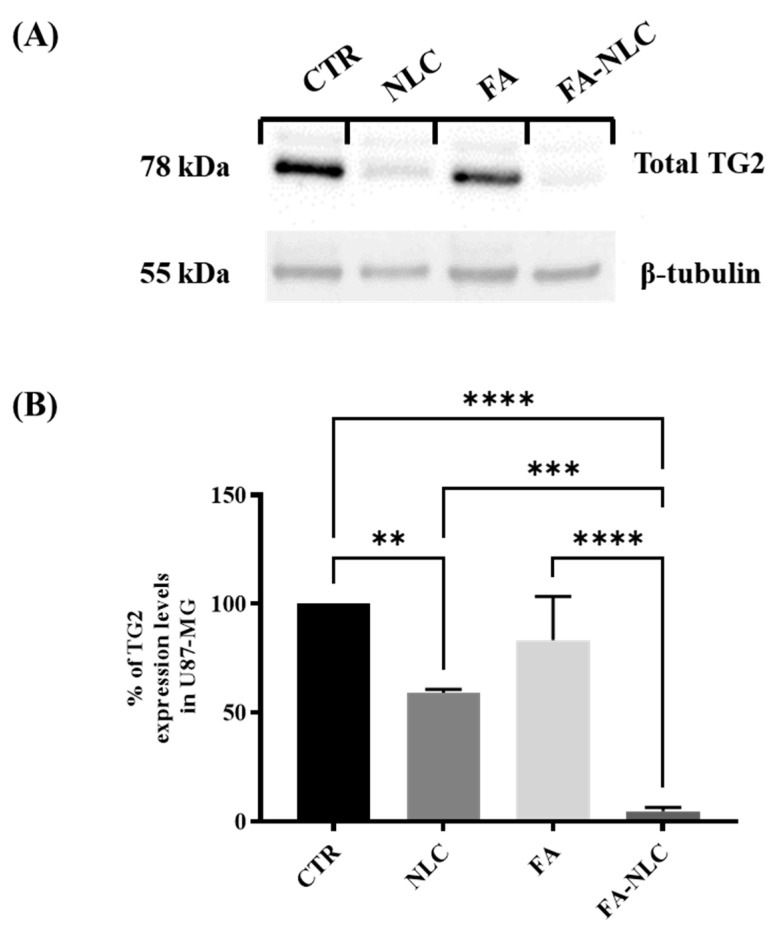
TG2 expression levels in U87-MG after 24 h treatment with blank NLCs, free FA, and FA-NLCs. Data were compared with the CTR. (**A**) Western blot analysis showing the expression levels of total TG2 in treated cells. β-Tubulin was used as house-keeping protein to normalize TG2 expression levels; (**B**) Histogram showing the densitometric analysis of the expression levels of TG2 at the different experimental conditions; Data reported in the histogram are value means of n = 3 different Western blots for TG2, after normalization with β-tubulin. Statistical analysis is evaluated through one-way ANOVA test. ** *p* = 0.0048 significant differences vs. control, *** *p* = 0.0007 significant differences vs. NLCs **** *p* < 0.0001 significant differences vs. control and FA-NLCs.

Figure 5 shows a representative immunoblot (Figure 5A) and the densitometric analysis (Figure 5B) of U87-MG cell cultures in all experimental conditions. TG2 expression appeared at high levels in control cells. The treatment of the cell cultures with blank NLCs induced a significant decrease in TG2 expression levels when compared with the control. The treatment of the cell cultures with free FA did not induce any significant decrease in TG2 expression levels when compared with the control. The effect was strongly evident in FA-NLCs-treated cells, and TG2 levels dramatically decreased (Figure 5A) when compared with the control. The densitometric analysis of TG2 expression levels (Figure 5B), normalized against β-tubulin, confirms the evidence obtained through Western blotting analysis. CLSM and Western blot analyses highlighted that the treatment with blank NLCs, free FA, and FA-NLCs differently modified TG2 localization and expression levels. In particular, when TG2 is localized in the nucleus, it plays a role in cell growth through its kinase activity; when it is localized in the cytosol, it exerts a pro-apoptotic effect. 

### 2.4. Involvement of TG2 in Cell Cycle Progression

**Figure 6:**
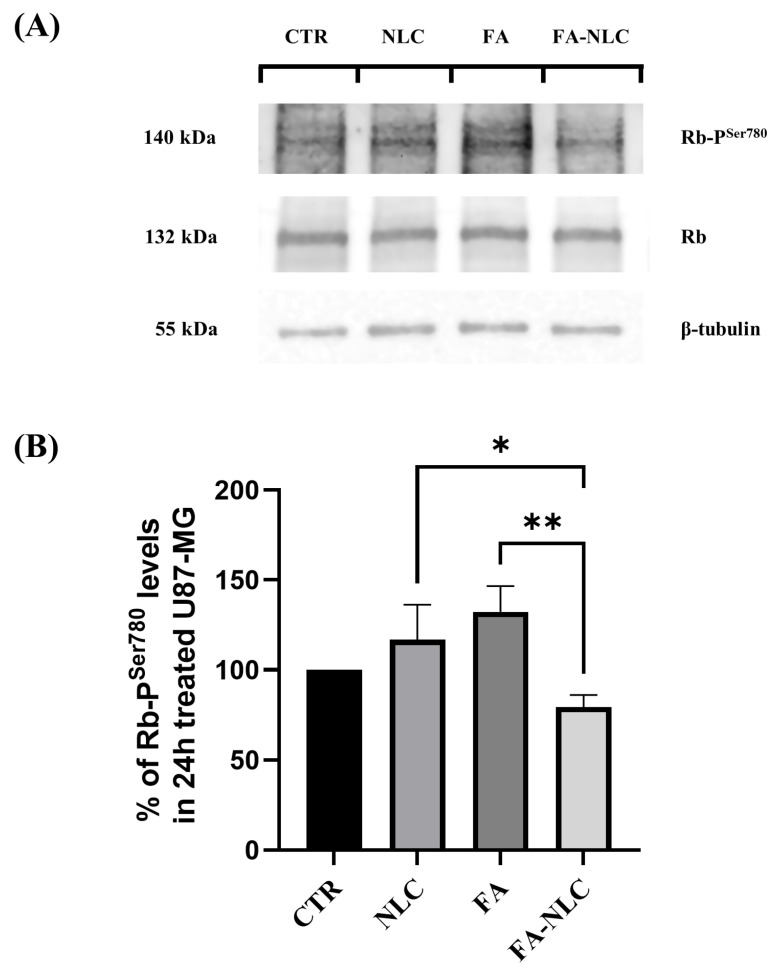
Analysis of Phospho-Ser780-Rb expression levels in U87-MG after 24 h treatment with blank NLCs, free FA, and FA-NLCs. (**A**) Western blot showing changes in phosphorylation of Rb. β-Tubulin is used as a housekeeping protein; (**B**) Histogram representing the expression levels of Phospho-Ser780-Rb at the different experimental conditions. Statistical analysis is evaluated through one-way ANOVA test. Results are expressed as the mean ± S.D. of the values obtained from n = 3 independent experiments performed in triplicate. * *p* = 0.0289 significant differences vs. NLCs, ** *p* = 0.0089 significant differences vs. FA-NLC.

To assess the involvement of the Rb-TG2-mediated phosphorylation (Ser780 residue), Rb unphosphorylated and phosphorylated, cyclin-D_1,_ and p53 expression levels in all experimental conditions were analyzed. Figure 6 reports the effect of the treatment for 24 h with blank NLCs, free FA, and FA-NLCs on U87-MG cell cultures. Blank NLC treatment induced a slight non-significant increase of Rb phosphorylation when compared with the control. The effect appeared more evident when the cells were exposed to free FA. A significant reduction in Rb phosphorylation in U87-MG treated with FA-NLCs was found when compared with the control. 

**Figure 7:**
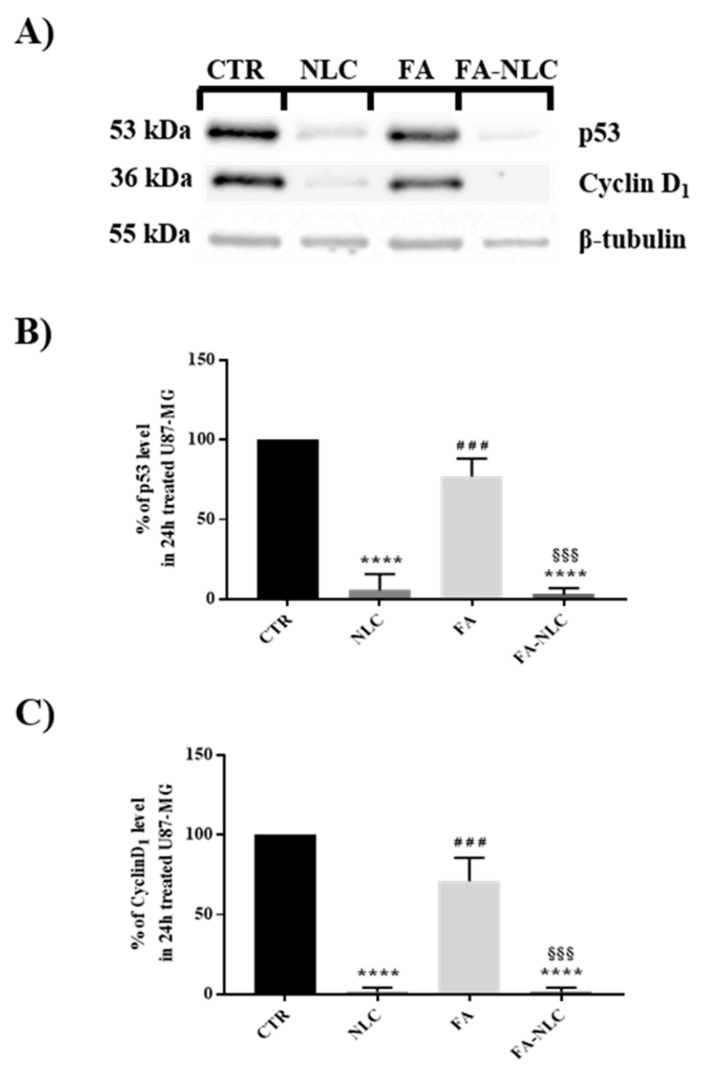
Analysis of p53 and cyclin-D1 expression levels in U87-MG after 24 h treatment with blank NLCs, free FA, and FA-NLCs. (**A**) Western blotting analyses showing changes in p53 and cyclin D_1_. β-Tubulin was used as a housekeeping protein; (**B**) Histogram representing the percentage of expression levels of p53; results are expressed as the mean ± S.D. of the values of four separated experiments performed in triplicate. **** *p* < 0.0001 significant vs. control; ### *p* = 0.0003 significant vs. NLC, §§§ *p* = 0.0002 significant vs. FA. (**C**) Histogram representing the percentage of expression levels of cyclin D1 at the different experimental conditions. Results are expressed as the mean ± S.D. of the values of n = 4 separated experiments performed in triplicate. Statistical analysis is evaluated through one-way ANOVA test. **** *p* < 0.0001 significant vs. control, ### *p* = 0.0001 significant vs. NLCs, §§§ *p* = 0.0001 significant vs. FA.

Cyclin D_1_ and p53 expression levels in U87-MG exposed for 24 h to blank NLCs, free FA, or FA-NLCs are shown in Figure 7. Blank NLCs induced a dramatic reduction of cyclin D_1_ expression levels when compared with the control. Free FA treatment only yielded a 30% reduction of cyclin D_1_ when compared with the control (Figure 7A,C). When the cells were exposed to FA-NLCs, a significant reduction in cyclin D_1_ was found. In particular, a reduction of 95–99% of the protein levels was observed when compared with the control. In parallel, the treatment of the cells with blank NLCs determined a 90–95% reduction in p53 expression levels when compared with the control (Figure 7A,B). In contrast, in the cell cultures exposed to free FA, a reduction of 20–23% of p53 expression was observed. In the U87-MG FA-NLCs treated cells, a significant decrease in p53 levels was found (90–95%) when compared with the control.

### 2.5. Apoptotic Pathway Evaluation

**Figure 8:**
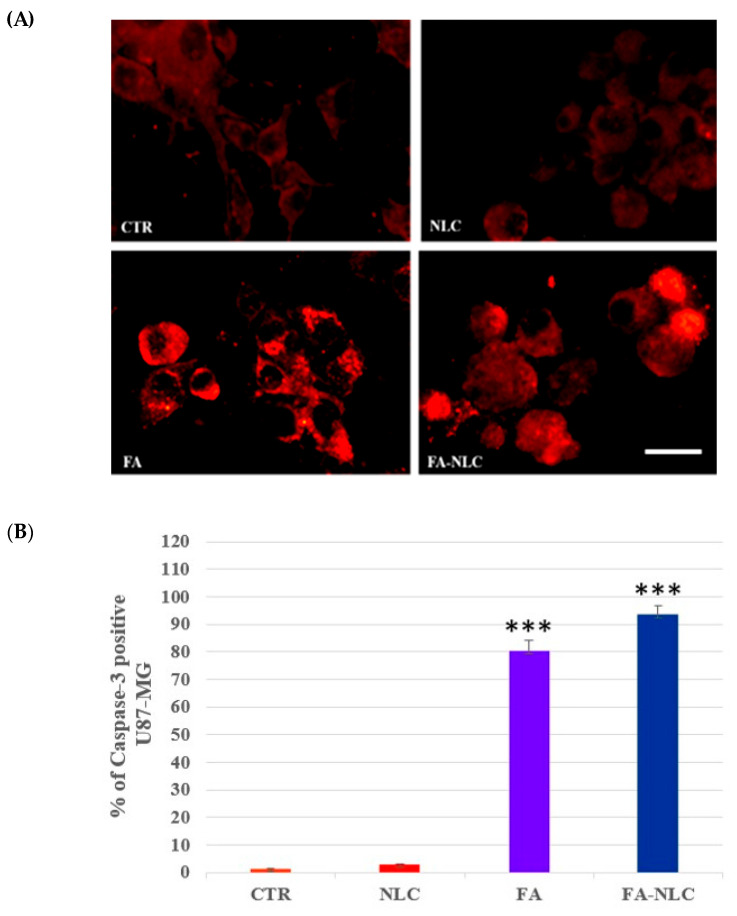
(**A**) Immuno-fluorescent positivity for caspase-3 in untreated U87-MG (CTR) and in treated cell cultures exposed for 24 h to blank NLCs (NLC), 36 µM free FA (FA), and 36 µM FA-NLCs (FA-NLC). Scale bars = 50 µm. (**B**) Immunofluorescence quantification and statistical analysis of caspase-3. Data are expressed as the mean ± S.D. of the values obtained from 10 fields/coverslips of the values of n = 3 independent experiments performed in triplicate. The statistic was performed through one-way ANOVA. *** *p* < 0.0001 CTR vs. FA and FA-NLC.

The effect of the treatment of U87-MG cell cultures with blank NLCs, free FA, or FA-NLCs on apoptotic pathway activation was assessed through the evaluation of caspase-3 and PARP-1 cleavage. A representative panel of pictures for caspase-3 cleavage from the different treatment conditions is reported in Figure 8A. In the control cells and in the blank NLC-treated ones, no significant positive cells for caspase-3 cleavage were found. The exposure of U87-MG cell cultures to free FA induced a significant enhancement of the positive cells for caspase-3 cleavage when compared with the controls. When the cells were treated with FA-NLCs, the positivity of the cells for caspase-3 appeared more evident. These observations were also supported by the changes in the cell morphology, presenting a typical apoptotic aspect. The positivity of the cells for caspase-3 in all experimental conditions was confirmed by the quantification and statistical analysis, as shown in Figure 8B.

**Figure 9:**
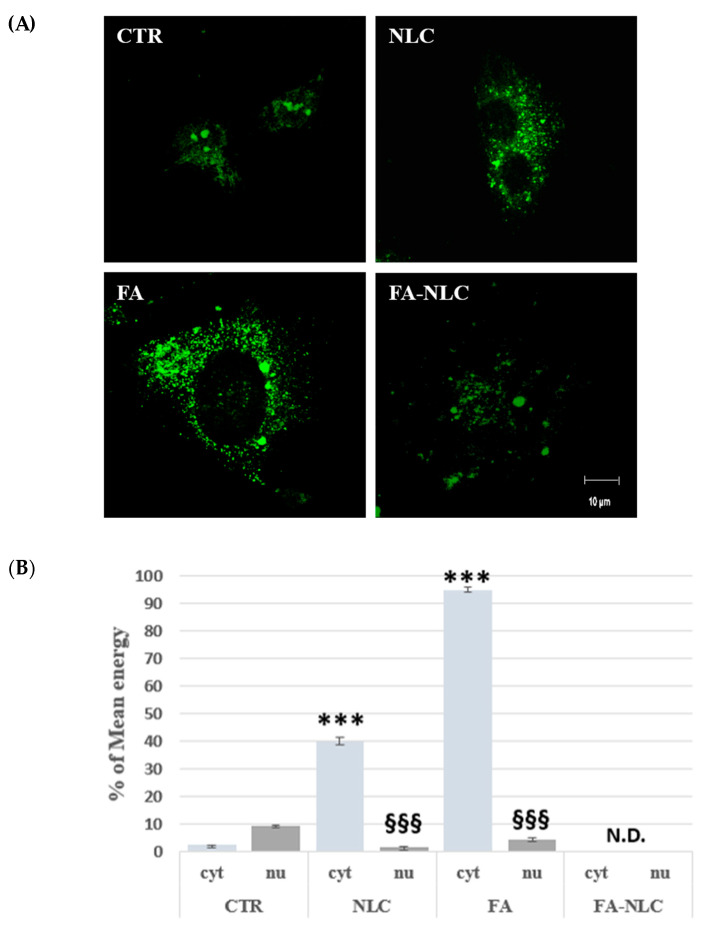
Panel (**A**) shows confocal images of U87-MG stained with PARP-1 antibody in the control condition (CTR) and after 24 h treatment with blank NLCs (NLC), 36 µM free FA (FA), and 36 µM FA-NLCs (FA-NLC). Cells were incubated with FITC-conjugated polyclonal antibody against PARP-1. Scale bar = 10 µm. (**B**) Histogram represents the CLSM mean energy of cytoplasmatic (cyt) and nuclear (nu) positivity for PARP-1 in the different experimental conditions. Data are expressed as the mean energy ± S.D. of the values obtained from 10 cells of the values of n = 3 independent experiments performed in triplicate. Statistic was performed through two-way ANOVA. *** *p* < 0.001 CTR (cyt) vs. NLC (cyt) and vs. FA (cyt); §§§ *p* < 0.001 CTR (nu) vs. NLC (nu) and vs. FA (nu); N.D. = not detectable.

Figure 9A shows representative CLSM images of PARP-1 localization and expression on U87-MG single cells incubated with FITC-conjugated polyclonal antibody against PARP-1. In the control condition, the protein was localized in the cytosol, in the nucleus, and in the nucleoli. When the cells were exposed to blank NLCs, a strong positivity for PARP-1 was observed that appeared prevalently localized in the cytosol when compared with the control. In particular, the protein appeared prevalently localized in the cytosol, even if a very low positivity for PARP-1 in the nucleus was found. The treatment of the cultures with free FA induced a strong positivity for PARP-1, and the protein appeared more evident in the cytosol. A low positivity in the nuclear compartment for PARP-1 was observed. The exposure of U87-MG to FA-NLCs showed a diffuse fluorescence relative to the cellular positivity for PARP-1 and the protein appeared almost undetectable both in the cytosol and in the nucleus. These results were accompanied by modifications of cell morphology, which showed a suffering aspect when compared with blank NLCs, free FA, and the control. No non-specific staining of cell cultures was revealed in controls incubated in the absence of the primary antibody. CLSM mean energy of cytoplasmatic (cyt) and nuclear (nu) positivity for PARP-1 in the different experimental conditions was performed through ImageJ Software (version 2.0.0-rc-69, 1.52p, free software downloaded using the Image Calculator function) (Figure 9B). These results confirmed our previous observation, indicating that the treatments of U87-MG cell cultures with blank NLCs, free FA, or FA-NLCs were able to activate the apoptotic pathway [22].

## 3. Discussion

GBM is characterized by high resistance to chemotherapy and radiation and exhibits a high propensity for recurrence [29,30,31,32,33]. It has been demonstrated that GBM and other cancers are related to altered redox homeostasis, which could represent a key point in the inhibition of tumor progression in terms of metastasis formation and drug resistance [18]. Previous observations highlighted that TG2 exerts an important role in GBM [32,33,34,35]. Furthermore, it has been reported that TG2 expression levels are strictly linked to intracellular ROS levels and that altering redox homeostasis activates alternative pathways that are responsible for drug/radio-resistance [18]. Recent studies indicate that the normalization of antioxidant capacity through treatment with natural antioxidant compounds capable of counteracting oxidative stress through TG2 signaling pathway modulation could represent a promising strategy for GBM therapy [18]. Many natural antioxidants, such as FA, possess anti-cancer, pro-apoptotic, and anti-proliferative properties, blocking cell cycle progression and also inducing autophagy [36,37,38]. Although it has numerous potential therapeutic properties, FA shows many disadvantages when administered in vivo, such as poor bioavailability, inability to cross lipophilic barriers, and extensive first-pass metabolism due to its low water solubility [39,40,41]. Therefore, we encapsulated it into NLCs in order to overcome these limitations and exploit its therapeutic properties. We chose these lipid nanoparticles as they have small-sized particles and are highly homogeneous and stable. Furthermore, they showed good systemic tolerability, high efficiency of drug encapsulation, and controlled release properties without any cytotoxic effect [42,43,44].

Previously, our research demonstrated that treatment with free FA of U87-MG human GBM cell line cultures was able to activate the apoptotic pathway. This effect was more evident when the cells were exposed to FA-NLCs due to the controlled drug release and its increased bioavailability [45,46,47,48,49]. Since the TG2 enzyme plays a dual functional role based on its cellular localization [50], it is important to understand how it influences cellular events [51,52,53]. In the cytosol, TG2 controls apoptotic processes through its transamidating activity; however, in the nucleus, it promotes cell growth through the phosphorylation of different proteins, such as Rb and p53 [54]. Herein, in this study what was assessed was the effect of blank NLCs, free FA, or FA-NLCs on TG2 localization and expression levels in U87-MG cell line cultures. Furthermore, to clarify the effect of FA on the role played by TG2 in apoptosis and in control of cell cycle progression through its kinase activity, un-phosphorylated and phosphorylated Rb Ser780 and cyclin-D1 were evaluated. In addition, FA effects were evaluated on p53 expression levels, protein up-regulated in cancers, and a substrate for TG2 kinase activity [7,8] in order to evaluate the effectiveness of this natural antioxidant. Since TG2 is also involved in the apoptotic pathway, we analyzed the effect of FA on the activation of apoptotic pathways, evaluating caspase-3 and PARP-1 cleavage. Firstly, we investigated the effect of blank NLCs, free FA, and FA-NLCs on cell viability using U87-MG, a human GBM cell line. The obtained results highlighted that treatment with blank NLCs did not influence U87-MG viability when compared with the control. This effect might be due to the use of biocompatible and biodegradable lipids used for NLC production. In contrast, when the cells were treated with FA, free or encapsulated in NLCs, a significant decrease in cell viability was observed. This effect was more evident in FA-NLC treatment, suggesting that NLCs represent a promising strategy for the delivery of FA and also improving its anti-cancer activity. As widely reported in the literature [27], NLCs are able to protect the encapsulated drug from external factors, enhancing its stability and bioavailability. Furthermore, they increase the drug’s cell uptake and, thus, its anti-cancer activity; the latter is probably due to the localization of TG2 in the cytosol. This result was confirmed by CLSM acquisition and Western blotting analysis performed in order to assess the intracellular localization and the expression levels of TG2 in U87-MG. In fact, in the free FA exposed, TG2 appeared expressed both in the cytosol and in the nucleus. In contrast, the treatment with FA-NLCs showed that the protein is exclusively localized in the cytosol. Furthermore, a slight reduction in TG2 expression has been observed compared to the control in the cells treated with blank NLCs. This could be due to the cationic composition of NLCs that interact with cell membrane components, sensitizing GBM cells to treatment [22].

To examine TG2 kinase activity in GBM and the effect of blank NLCs, free FA, and FA-NLCs, the phosphorylation of Rb at the Ser^780^ residue, p53, and cyclin-D_1_ expression levels were also evaluated. We observed that blank NLCs induced a slight increase in Rb phosphorylation when compared with the control. In contrast, both FA and FA-NLC treatments led to a significant reduction in Rb phosphorylation when compared with the control. These data demonstrate that the treatment with free FA and FA-NLCs was able to reduce TG2 kinase activity. This effect was also supported by the significant reduction in cyclin D_1_ and p53 expression levels induced by the treatment of U87-MG cell line cultures. In addition, the treatment with FA-NLCs dramatically affected the positivity of the cells for TG2 in the nucleus and in the nucleoli. In addition, to verify if FA was able to activate the apoptotic pathway TG2-mediated, we evaluated the assessment of caspase-3 and PARP-1 cleavage [55,56,57,58]. In particular, we investigated if free FA and FA-NLC treatments were able to stimulate PARP-1-dependent cell death. We found that the exposure of the cells to FA, free and loaded into NLCs, showed a different positivity for PARP-1 of the cells and a different localization depending on treatment type. In the control cells, the protein was localized in the cytosol, in the nucleus, and in the nucleoli. The exposure to the blank NLCs induced a translocation of PARP-1 from the nucleus to the cytosol, where it showed a strong positivity. When the cultures were treated with free FA, a strong positivity for PARP-1 in the cytosol was observed, while the exposure to FA-NLCs showed a diffuse fluorescence relative to the cellular positivity for PARP-1 and the protein appeared almost undetectable both in the cytosol and in the nucleus. 

This evidence suggests that the treatment with free FA and, in particular with FA-NLCs, might induce the translocation of the PARP-1 from the nucleus to the cytosol and its fragmentation, mediated by caspase-3, which might be responsible for the protein inactivation and consequent DNA damage. These results demonstrate that the treatment of the cells with free FA and FA-NLCs induces a remarkable decrease in TG2 expression protein in the nuclear compartment. This observation highlights that the treatment of the cultures of GBM with FA, free and loaded into NLCs, is able to block the role played by TG2 in cellular growth and proliferation. 

**Figure 10:**
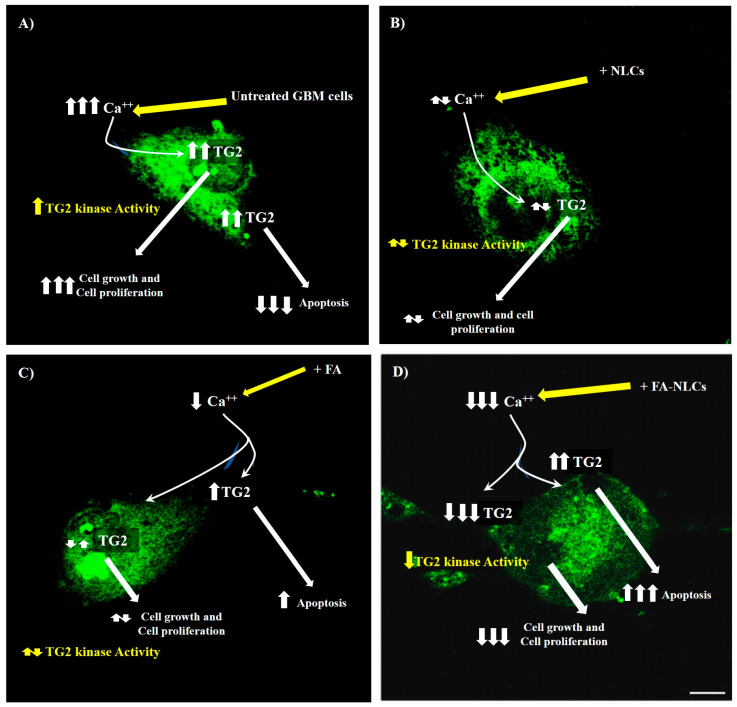
Drawing regarding the effects of U87-MG treatments with blank NLCs (**B**), free FA (**C**), and FA-NLCs (**D**) on TG2 expression levels compared to the control (**A**). ↑ = increase, ↑↑ = significant increase, ↑↑↑ = remarkable increase, ↑↓ = unchanged, ↓ = decrease, ↓↓↓ = remarkable decrease.

Our findings suggest that TG2 might play different roles in the human GBM cell line depending on intracellular calcium levels [13]. We hypothesize that, based on the treatment type, the protein could undergo modifications both in intracellular localization and expression levels. In the untreated GBM cells, the protein expression appeared at high levels in the nuclei, nucleoli, and cytosol. This effect might be related to the high levels of Ca^2+^ and intracellular ROS typical of cancer (Figure 10A). Surprisingly, in the blank NLC-exposed cells, TG2 is prevalently localized in the cytosol. This result could be due to the cationic charge of NLCs, which might reduce the intracellular calcium levels and, consequently, TG2 expression and its localization (Figure 10B). In free FA-treated cells, TG2 is localized in the nucleus and in the cytosol, although the cells appeared distressed and underwent apoptosis, as revealed by caspase-3 and PARP-1 cleavage. These results might support the hypothesis that the pro-apoptotic effect on TG2 is exerted by free FA treatment (Figure 10C). The effect is more evident in GBM cells exposed to FA-NLCs, which induce a strong reduction in TG2, probably counteracting intracellular Ca^2+^ levels able to restore p53 and cyclin D_1_ expression to normal cells. This effect might be related to a significant reduction in TG2 kinase activity (Figure 10D).

## 4. Materials and Methods

### 4.1. Materials

Cetyl Palmitate (Cutina CP) was from BASF Italia S.p.A (Cesano Maderno, MB, Italy). Oleth-20 (Brij 98), isopropyl stearate (IPS), and Gliceryl Oleate (Tegin O) were from A.C.E.F. S.p.a. (Piacenza, Italy). Ferulic acid and Ceteth-20 (Brij 58) were provided by Fluka (Milan, Italy). Didodecyldimethylammonium bromide (DDAB), leupeptin, aprotinin, phenylmethylsulfonyl fluoride (PMSF), EDTA, EGTA, Sodium Dodecyl Sulfate (SDS), and phosphatase inhibitor cocktail II were from Sigma-Aldrich (Milan, Italy). Sodium Pyruvate, Isoceteth-20 (Arlasolve 200), 3(4,5-dimethyl-thiazol-2-yl)2,5-diphenyl-tetrazolium bromide (MTT), Dimethyl sulfoxide (DMSO), Lab-Tek II Chamber-Slide Systems, tetrarhodamine isothiocyanfluorescein isothiocyanate (FITC)-conjugated anti-mouse IgG poly-clonal antibody, tetramethylrodamine isothiocyanate (TRITC), and other analytical chemicals were purchased from Sigma–Aldrich (Milan, Italy). TG2 was provided by Abcam (Milan, Italy), while goat-polyclonal-Rb and its phosphorylated derivative were obtained from Santa Cruz Biotechnology (Heidelberg, Germany). Regenerated cellulose membranes (Spectra/Por CE; Mol. Wet. Cut off 3000) were from Spectrum (Los Angeles, CA, USA). Water, acetic acid, and methanol were of LC grade and purchased from Merck (Milan, Italy). All other reagents were of analytical grade. The U87-MG human glioblastoma cell line was from the Cell Bank Interlab Cell Line Collection (Genova, Italy). Trypsin, antibiotics, non-essential amino acids, health inactivated Fetal Bovine Serum (FBS, GIBCO), Phosphate Buffer Saline solution (PBS), Normal Goat Serum (NGS, GIBCO), Dulbecco Modified Eagle Medium (DMEM) with 2 mM Glu-taMAX (GIBCO), WesternBreeze Chemiluminescent Western Blot Immunodetection Kit were from Invitrogen (Milan, Italy). Bicinchoninic acid Protein Assay Kit was from Pierce (Thermo Fisher Scientific, Milan, Italy). Mouse monoclonal antibody anti-TG2 and rabbit monoclonal antibody anti-β-Tubulin were from Cell Signaling Technology (EuroClone, Milan, Italy). Mouse monoclonal antibody against PARP-1 was from Trevigen (Tema Ricerca s.r.l., Castenaso, Italy).

### 4.2. NLC: Preparation and Characterization

NLCs were prepared by the phase inversion temperature method, an eco-friendly technique previously described [28]. The aqueous phase, added with oleth-20 (8.7% *w*/*w*) and glyceryl oleate (4.4% *w*/*w*), and the lipid mixture (5% *w*/*w*) composed of cetyl palmitate, IPS, and the cationic lipid DDAB (0.5% *w*/*w*), were separately heated at ~80 °C. Then, the aqueous phase was added drop by drop, at constant temperature and under agitation, to the oil phase. The obtained mixture was cooled to 60 °C, successively subjected to three thermal cycles, and then cooled to room temperature under slow and continuous stirring. FA-NLCs were formulated following the same protocol by adding the drug (0.7% *w*/*w*) to the oil phase.

The mean particle size, polydispersity index (PDI), and zeta potential (ZP) values of NLC formulations were obtained using Zetasizer Nano S90 (Malvern Instruments, Malvern, UK) [27]. Before measurements, each sample was diluted (1:200) with ultra-purified water.

The percentage of the encapsulated drug (EE%) and the drug loading capacity (LC%) were obtained through ultracentrifugation for 1 h at 1000 rpm (Beckman model J2–21 Centrifuge). The obtained pellet was dissolved in methanol, filtrated (0.22 µm), and analyzed using a Varian Prostar 230 HPLC (Varian, Milan, Italy) fitted with a reversed-phase C18 column (Symmetry, 4.6 cm ×15 cm; Waters, Milan, Italy). The mobile phase consisted of methanol/CH_3_COOH (60:40 *v*/*v*). The FA calibration curve was constructed in the range of 0.1–100 µg/mL (R^2^ = 0.9997). Possible lipid interferences were also studied. In vitro release experiments were performed using Franz-type diffusion cells [26]. Cryogenic transmission electron microscopy (Cryo-TEM) was used to characterize FA-NLCs using the procedure described in our previous study [59].

### 4.3. Glioblastoma Cell Line Cultures

A U87-MG cell line was maintained in DMEM containing 10% FBS, 1% Non-Essential Amino Acids, penicillin (50 U/mL), streptomycin (50 µg/mL), 2 mM GlutaMAX, and 1 mM Sodium Pyruvate seeded in 25 cm^2^ flasks at a final density of 2 × 10^6^ cells and incubated at 37 °C in a humidified atmosphere containing 5% CO_2_. Change medium was performed every 2 or 3 days. When the cultures were about 85–90% confluent, cells were seeded in 100 mm Ø dishes or Lab-Tek II Chamber-Slide and incubated at 37 °C in a humidified atmosphere containing 5% CO_2_.

### 4.4. Treatment of U87-MG Glioblastoma Cell Line Cultures

U87-MG cultures were exposed for 24 h to the different treatments: PBS, DMSO, free FA, blank NLCs, and FA-NLCs. The free FA, blank NLCs, and FA-NLCs stock solutions were prepared as follows: free FA (7 mg/mL) was diluted in DMSO, while blank and FA-NLCs (50 mg/mL referred to the solid lipid, FA concentration 0.7% *w*/*w*) were diluted in PBS [22]. For each test, the suitable aliquot from each stock solution was added to the culture medium in order to obtain a 36 µM FA final concentration, corresponding to a final NLC concentration of 0.5 mg/mL. Two groups of cells were treated with PBS, used to dilute NLC, or with DMSO, having a final concentration of 0.01% *v*/*v*, used to solubilize FA. Since both cell groups respond similarly to PBS or DMSO, we used them as control (CTR) groups.

### 4.5. MTT Bioassay

Cell viability was assessed by the MTT Test to assess the FA concentration in untreated (control) U87-MG with free FA and loaded into NLC and to establish the exposure time of the cell lines. Cells seeded in a 96-multiwell plate, after the treatments, were exposed to 20 µL of MTT stock solution, 5 mg/mL PBS, in 200 µL medium per well [60]. After 2 h incubation, the medium from each well was removed and replaced with 100 µL of DMSO. The optical density from each well was measured through a microplate reader at λ of 570 nm. Results are reported as a percentage of the control, taken as 100%, to normalize the different obtained values.

The effective concentration of FA used to treat U87-MG in this assay and in the successive was 36 µM, as we had established in a previous publication [28]. PBS and DMSO-treated cell groups were used as controls.

### 4.6. Immunocytochemical Assay

U87-MG cell lines were seeded in Lab-Tek II Chamber-Slide Systems at the final density of 0.5 × 10^5^ cells/mL and incubated at 37 °C in 5% CO_2_–95% air. PBS, DMSO, NLC, 36 µM free FA, and 36 µM FA-NLC were used to treat 80% confluent cells for 24 h. After the treatments, the cells were fixed for 20 min with 4% paraformaldehyde, washed with PBS, and cell membranes were permeabilized with 5% normal goat serum (NGS) in PBS containing 0,1% Triton X-100 at room temperature for 30 min to block non-specific sites [10]. Subsequently, they were incubated overnight at 4 °C with anti-TG2 (diluted in PBS 1:100), anti-caspase-3 (diluted in PBS 1:200), or anti-PARP-1 (diluted in PBS 1:100) mouse monoclonal antibodies. Thereafter, the cells were washed with PBS and incubated for 2 h with anti-mouse IgG conjugated polyclonal antibody with fluorescein isothiocyanate (FITC) (1:64 in PBS). Finally, the cells were washed with PBS and mounted in PBS/glycerol (50:50). The slides were examined for caspase-3-positive cells with a fluorescent microscope (Leica Microsystems, Wetzlar, Germany). In particular, to assess TG2 and PARP-1 localization in the cytosol and into nuclear compartment, cells were incubated with mouse monoclonal antibody against TG2 or PARP-1 and, then, were incubated with anti-mouse IgG conjugated polyclonal antibody with fluorescein isothiocyanate (FITC) (1:64 in PBS). TG2 and PARP-1 positive cells were analyzed by Confocal Laser Scanning Microscope (CLSM, LSM-510 Meta, Zeiss, Germany). CLSM images were acquired through an Apo 63 X/1.4 oil immersion objective and the Argon (λ = 488 nm) laser (Leica Microsystems, Wetzlar, Germany). Images were acquired at 1024 × 1024-pixel resolution and were processed using the 2009 Zen (software version n. 5.5.0.452) supplied with the confocal microscope ZEISS (ZEISS, Milan, Italy). To check for non-specific staining of U87-MG, the primary antibody was omitted in control incubations, and no stain was observed [10]. The fluorescence was performed by analyzing the intensity energy 257 of channels from multiple regions, normalizing them to the background through the ImageJ Software (version 2.0.0-rc-69, 1.52p, free software downloaded using the Image Calculator function). Statistical analysis showing the positivity for TG2 or PARP-1 was performed on ten single cells for each microscopy field for n = 3 independent experiments.

### 4.7. Western Blotting Analysis

Experimental treated cells were pelleted and homogenized in 1X lysis buffer [61]. Protein concentration was determined by using the BCA method (BCA Protein Assay Kit, Pierce). A total of 50 µg of total proteins were electrophoresed through 4–15% pre-casted SDS-PAGE (BioRad Laboratories S.r.l., Milan, Italy). The obtained filters were incubated with the specific primary antibodies (dilution 1:1000) mouse monoclonal against TG2, goat-polyclonal-Rb, goat-polyclonal-Phospho-Rb, goat-polyclonal-p53, rabbit-monoclonal-cyclin D_1_, and rabbit-monoclonal against β-tubulin. Anti-rabbit and anti-mouse secondary antibodies linked to alkaline phosphatase (AP) and anti-goat secondary antibodies linked to horse radish peroxidase (HRP; dilution 1:10,000) were used. WesternBreeze Chemiluminescent Western Blot Immunodetection Kit (Invitrogen) was used to reveal immunobands. The immuno signals were detected through the VersaDoc Imaging System (Bio-Rad Laboratories Srl, Italy) and evaluated by densitometric analysis using the Quantity One software (version number: 4.6.7).

### 4.8. Statistical Data Analysis

Each analysis was executed in triplicate for each experimental condition. Three or four independent experiments were conducted. Data obtained were statistically analyzed using one-way analysis of variance (ANOVA) followed by a post hoc Holm–Sidak test or by Tukey’s multiple comparisons test to evaluate significant differences among experimental groups. Data were expressed as mean ± S.D. quantification analysis, as mean ± S.E. Statistical significance is reported in figure captions.

## 5. Conclusions

Our findings demonstrate that the treatment with blank NLCs, free FA, and FA-NLCs in a U87-MG cancer cell line differently modified TG2 localization and expression levels. Free FA and, in particular, FA loaded in NLCs are able to inhibit TG2 kinase activity, disrupting redox homeostasis and activating intrinsic and extrinsic apoptotic pathways. Furthermore, it led to an increase in p53 and cyclin D_1_ expression levels, stimulating its anti-proliferative role. Since tumor cells are closer to the threshold of ROS toxicity, the alteration of redox homeostasis may be a potential target for treating cancer. This could be achieved through antioxidant administration, such as FA, which inhibits TG2 expression by stimulating ROS production and altering redox homeostasis beyond the threshold of ROS toxicity and, thus, tumor cell death.

The promising findings of the present work suggest future research directions to test the in vivo experimental model for the efficacy of FA-NLCs in the treatment of GBM.

## Data Availability

The data used to support the findings of this study are available from the corresponding author upon request.
